# Measurement of Body Condition in a Common Carabid Beetle, *Poecilus cupreus*: A comparison of Fresh Weight, Dry Weight, and Fat Content

**DOI:** 10.1673/031.013.0601

**Published:** 2013-01-24

**Authors:** Michal Knapp, Jana Knappová

**Affiliations:** 1 Department of Ecology, Faculty of Environmental Sciences, Czech University of Life Sciences Prague, Kamýcká 129, Praha 6 - Suchdol, 165 21, Czech Republic; 2 Institute of Botany, Academy of Sciences of the Czech Republic, Průhonice, 252 43, Czech Republic

**Keywords:** Coleoptera, Carabidae, fitness, insect, lipid content, nutritional condition, structural body size

## Abstract

Because of its direct consequences on reproductive success, body condition is an often-studied individual trait in insects. Various studies on insects use disparate methods to assess “body condition.” However, it is doubtful that the results obtained by disparate methods are comparable. In this study, the body conditions *of Poecilus cupreus* (Linnaeus) (Coleoptera: Carabidae) from eight sites were compared based on the following commonly used variables: (i) fresh weight, (ii) dry weight, and (iii) fat content. All of these variables were corrected for structural body size. Moreover, the effects of using the following ways of assessing structural body size were examined: (a) one size measurement (length of elytron, which is commonly used in beetles), and (b) three size measurements (length of elytron, width of pronotum and length of hind femur). The results obtained using the various estimations of body condition (i, ii, iii) varied significantly. Therefore, studies employing distinct body measurements to assess body condition are not comparable to each other. Using multiple structural size measurements in body condition analyses is better than the common practice of using only one size measurement. However, in the present study, results provided by both methods differ only slightly. A recommendation on the use of terminology in studies on body condition is introduced.

## Introduction

Information on the body condition of animals (in terms of energy reserves) is very important in ecological research. Individuals in better body condition have higher longevity in unfavourable conditions (Petersen 1999), higher fecundity (Moya-Larano 2002; Barone and Frank 2003), and higher mating success (Cotton et al. 2006). Therefore, body condition can be considered to be an indicator of health and potential reproductive success of individuals. Moreover, body condition can be used as an indicator of habitat quality for some species (e.g., Bommarco 1998a; Barone & Frank 2003). Such indirect evidence of habitat quality is very useful, especially for arthropods, because there is limited information on the ecological requirements of most species.

Body condition is commonly expressed as an indirect estimate of nutrient storage, and is usually reported as some measure of body mass. Because larger individuals achieve a higher mass than smaller ones, mass has to be corrected for individual body size to express body condition. Typically, structural body size (Moya-Larano et al. 2008) is used for this purpose. Structural body size provides a measure of size that does not vary during adulthood in relation to nutrient intake. In insects, structural body size is generally measured using the sizes of sclerotised body parts (e.g., head, pronotum, etc.).

Measurement of either live weight or the volume of the body parts used for storage of energy reserves (Moya-Larano et al. 2008) is usually the only available method for assessing body condition in vertebrates and live insects. However, in dead insects, one can also measure dry weight or even directly assess the fat content (Nedved and Windsor 1994; Östman 2005). Moreover, measurement of structural body size in live specimens is often difficult and inaccurate, whereas dead specimens may be measured quite precisely. Because it is often necessary to kill insect specimens in the course of field studies, either due to the sampling method or as a necessity for identification of species, assessing body condition in dead specimens is a quite common practice.

There is ongoing debate regarding the optimal method of statistical analysis of body condition data, and in particular, how to apply the correction for structural body size (Jakob et al. 1996; Garcia-Berthou 2001; Green 2001; Freckleton 2002; Peig and Green 2009). The most frequently used methods in arthropods, ratio index (sometimes called “condition factor”; e.g., Barone and Frank 2003) and analysis of ordinary least squares residuals (e.g., Öberg 2009), do not seem to be optimal. A more appropriate alternative seems to be multiple regression analysis, in which variables representing structural body size are used as covariates in the model (Garcia-Berthou 2001; Freckleton 2002).

In contrast to the numerous guides on statistical analysis of body condition, there is a lack of literature on how to appropriately measure body condition and structural body size. Various studies use disparate methods to assess body condition of arthropods (Barone and Frank 2003; Östman 2005; Frank et al. 2007; Öberg 2009), but the term “body condition” or “nutritional condition” is used without specifying the method used to assess it. Therefore, the present study is interested in assessing how comparable the results obtained with the various methods are, and whether these diverse methods are likely to qualitatively affect study conclusions.

To obtain a suitable dataset for comparison of the various measures of body condition, data from one year of a long-term field study investigating temporal variability in body condition of *Poecilus cupreus* (Linnaeus) (Coleoptera: Carabidae) (Knapp M., unpublished data) were used. The performance of three mass variables (fresh weight, dry weight, fat content) were compared while analyzing the effects of field, boundary type, and gender. Moreover, the effects of using only one versus several measures of structural body size in body condition analyses were compared. Because fat extraction and multiple measurements of body size are time-consuming, the necessity of these procedures is of particular interest.

## Materials and Methods

### Study species and sites

*P. cupreus* is a common carabid beetle with West Palearctic distribution. This species inhabits a wide range of open habitats, including arable fields (Thiele 1977; Hurka 1996). Newly-emerged adult *P. cupreus* are active from late summer to autumn, and after overwintering (outside of arable fields) they reproduce in early summer, frequently in arable fields (Wallin 1986).

The specimens used in the present study were collected in four fields near Prague - Suchdol, Czech Republic (50° 8′ N, 14° 21′ E). In each field, two sites at the field edge that differed in adjacent habitat, having either grass or forest, were sampled. Beetles were collected in the second half of October 2009, shortly before overwintering. When possible, 30 specimens (15 males and 15 females) from each site were collected. Live beetles were killed by freezing and were stored in a freezer at -20° C until processing in the laboratory. Freezing seems to be an optimal method for storage of specimens subsequently analyzed for body condition (Knapp 2012).

### Size, weight, and fat content measurements

Immediately after thawing, each beetle was cleaned to remove particles of soil, and the measurement of fresh weight was performed. Beetles were subsequently dried at 60° C for 48 hours, and then dry weight was measured. For fat extraction, each specimen was individually placed in an Eppendorf® tube (1.5 mL) and submerged in a 1:1 (vol:vol) mixture of diethyl ether and chloroform for 72 hours (Nedved and Windsor 1994; Östman 2005). Then, the beetles were dried again for 48 hours and subsequently weighed (lean dry weight). Fat content was calculated as the difference between the dry weight and lean dry weight. All weights were measured with a Sartorius® analytical balance to a precision of 10^-5^g.

Structural body size was measured as: “length of elytron” (the longest distance from the elytron apex to the elytron base); “width of pronotum” at its widest part; and “length of femur” of the hind leg. All proportions were measured using a digital calliper to a precision of 0.01 mm.

### Statistical analyses

To compare results obtained by different variables representing body condition, generalized linear models with gaussian distribution of errors (GLM, analogous to nested analysis of covariance) were performed. The effects of field, boundary (nested within field), and gender on body condition (measured in various ways) were investigated. For purposes of the present study, no additional information (e.g., field area or heterogeneity of surrounding landscape) was associated with sites where beetles were collected. Only the presence of the effects of field and boundary (yes or no, not why) and the difference of these effects among employed variables were of interest. Therefore, individual beetles (not site means) represent independent data points for the GLM. Response variables (fresh weight, dry weight, fat content) were log-transformed prior to analyses. For each response variable, two models that differed in covariates representing the structural body size correction used (defined below) were examined.

For each of six models (representing the combination of fresh weight, dry weight, and fat content with two ways of measuring structural body size), analysis started with the full model consisting of covariate(s), independent variables (field, field × boundary interaction, gender), and interactions of gender with other independent variables. To obtain the final models, backward stepwise selection based on deletion tests (F-test) was used. The following covariates were used in the models: (i) length of elytron, or (ii) length of elytron, width of pronotum, and length of femur. In the latter case, forward stepwise selection among these covariates was performed at the first step.

**Table 1.**
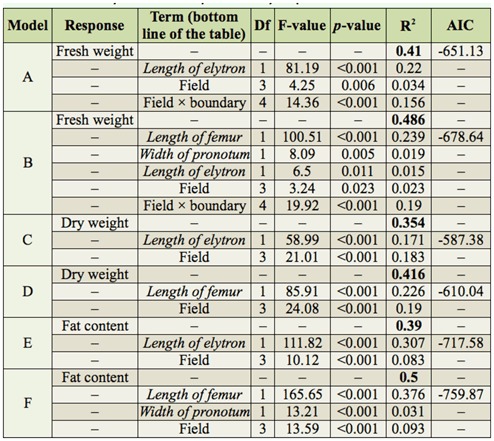
Final generalized linear models investigating the effects of field, boundary type (nested within field), gender, and their interactions on body condition of *Poecilus cupreus* for all combinations of surrogates of body condition (fresh weight, dry weight, fat content) and two methods of measuring structural body size (single structural measurement, multiple structural measurements). AIC scores enable the comparison of models with the same response variable (e.g., A and B; lower AIC scores indicate a superior model). Terms in italics indicate covariates included in models as corrections for structural body size. In models B, D, F, covariates were selected according to forward stepwise selection from length of elytron, width of pronotum, and length of femur. R^2^ values in bold represent total variability in the response variable explained by a particular model. Regular R^2^ values represent the proportion of variability in data explained by a particular term.

To display significant differences in body condition (measured as fresh weight, dry weight, or fat content) among sites, post-hoc tests for GLM (Tukey's HSD) implemented in package “multcomp” were used (Hothorn et al. 2008). All analyses were performed in R 2.11 (R Development Core Team 2010).

## Results

In total, the body condition of 227 beetles (115 males and 112 females) originating from eight sites (four fields × two boundary types) was analysed. Body condition measured as fat content or dry weight was significantly affected by the field of origin. However, body condition measured as fresh weight was significantly affected by field and boundary type (interaction field × boundary in the present model; Table 1). There was no effect of gender on body condition measured as fresh weight, dry weight, or fat content.

**Figure 1.**
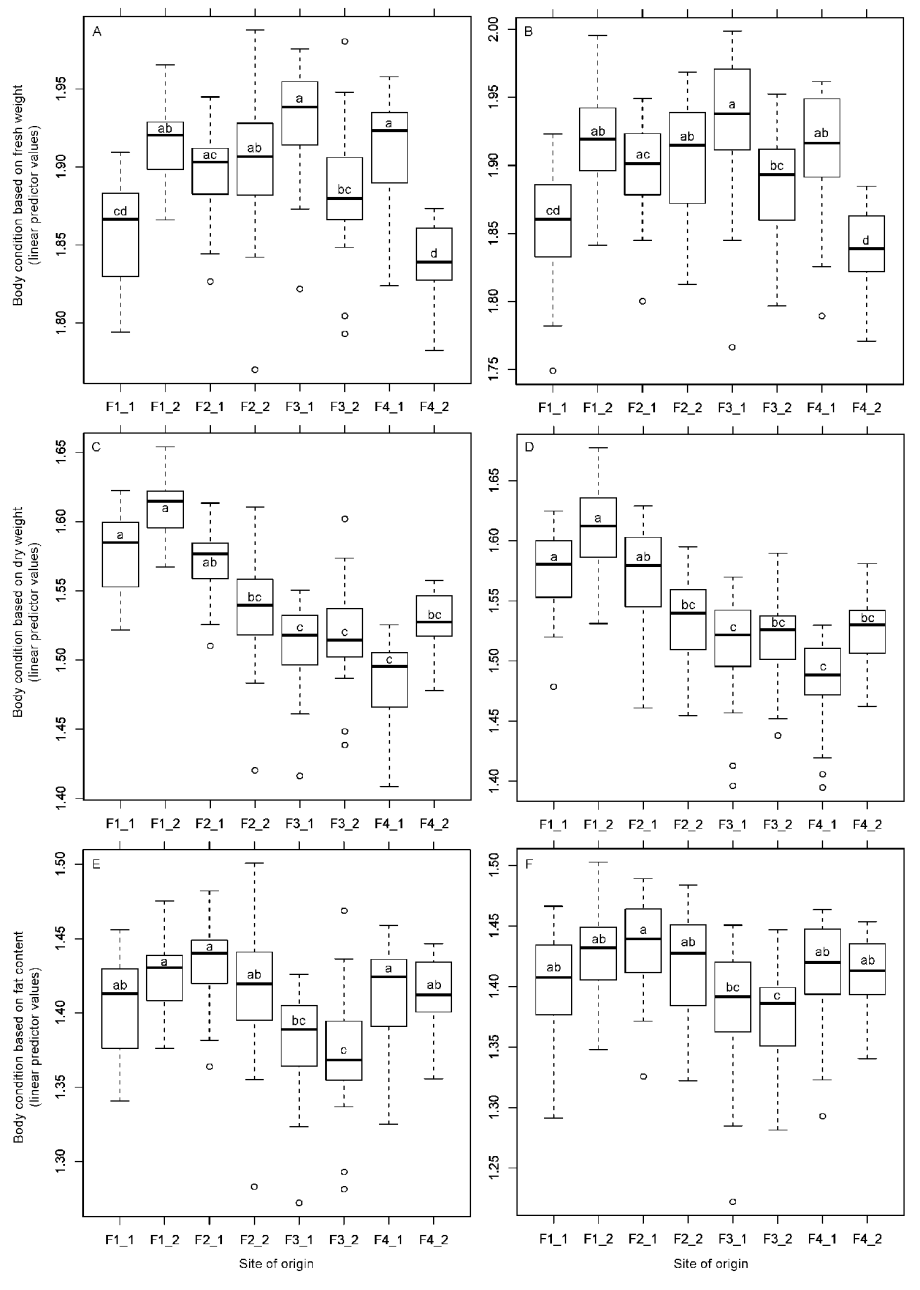
Comparison of body condition of *Poecilus cupreus* originating from various sites based on measurement of fresh weight, dry weight and fat content, in combination with two ways of measurement of structural body size. Within a particular figure, sites differing in body condition of collected beetles do not share the same letters (Tukey's HSD: *p* < 0.05). In figures A, C, and E, length of elytron was used as covariate, whereas in figure B, length of hind femur, width of pronotum, and length of elytron were used as covariates. In figure D, length of hind femur was used, and in figure F, length of hind femur and width of pronotum were used for correction on structural body size (according to forward selection procedure; Table 1). Linear predictor values produced by “plot.cld” function in the package “multcom” are presented (Hothorn et al. 2008). High quality figures are available online.

Detailed comparison of body condition among the sites of origin revealed that body condition varied among sites in distinct patterns that differed depending on the method of measurement (fresh weight, dry weight, fat content; Figure 1). Dry weight and fat content did not provide comparable results, although there is a relatively close relationship (correlation) between fat content and dry weight of beetles within particular sites (r-value between 0.84 and 0.96).

For all variables (fresh weight, dry weight, fat content), structural body size based on multiple body size measurements (length of elytron, width of pronotum, length of hind femur) performed better in comparison to commonly used one size measurement (length of elytron; see AIC scores and R^2^ in Table 1). For the analysis of body condition, however, the use of multiple structural size measurements seems to produce results that are only slightly different to the use of only one structural size measurement (Figure 1). Overall, length of hind femur performed as the superior single measure of structural body size (see rank of covariates used in models B, D, and E in Table 1).

## Discussion

Body condition is an often-studied individual trait in arthropods (Colinet et al. 2006; Frank et al. 2007; Öberg 2009). However, there is no standard method for measuring body condition, so various studies use different methods to assess this characteristic. Some studies directly measure fat content (e.g., Östman 2005), while others measure dry weight (e.g., Frank et al. 2007) or even fresh weight (e.g., Barone and Frank 2003). The present study found that patterns obtained by using different measurements were not consistent. Therefore, the method used to assess body condition in a particular study has to be emphasized to clarify what is being referred to as “body condition.”

In carabids, high energetic demands of pupation connected with production of defensive secretions against predators result in newly emerged adults with often completely exhausted energy reserves (Giglio et al. 2009). Fresh weight and fat content generally increase in adult carabids in time, and this increase is especially apparent in the first few weeks after eclosion (Chaabane et al. 1997). Moreover, Bommarco (1998b) illustrated the importance of food consumed by adult *P. cupreus* on reproduction success (fecundity). Therefore, body condition is constituted mainly during the adult stage in carabids and is given by environmental conditions, especially by food quality and quantity, supporting the idea that the body condition of an individual reflects habitat quality (Barone and Frank 2003).

Rolffand Joop (2002) stated that dry weight is superior to fresh weight in body condition analyses in damselflies. We agree and assume that fat content, assessed by direct fat extraction and corrected for structural body size, is an even better estimate of body condition (in terms of energy reserves) because fat stored in the fat body serves as the main reservoir of energy for insects (Arrese and Soulages 2010). Only a small fraction of lipids in arthropods have a function other than storage (Lease and Wolf 2011). By contrast, dry weight measures not only lipids but also the content of other substances in the body, e.g., protein. Although these substances may be of substantial importance for the fitness of insects (Bennett et al. 2005), they are not related directly to the energy reserves of individuals. As a measure of body condition, one can expect fresh weight to perform differently in comparison to fat content or dry weight because there is high variation in the water content of arthropods depending on environmental conditions (Nedved and Windsor 1994; Bennett et al. 2005). Similarly to protein, water content can be of high importance in some conditions, e.g., desert or arctic environments (Block and Convey 2001; Cloudsley-Thompson 2001), but it is not directly related to energy reserves. However, body condition is often used as a general surrogate for fitness (not only for energy reserves), and the fitness of an animal does not have to be connected exclusively to energy reserves (e.g., Kumano et al. 2010). For this purpose, it is necessary to investigate the relationship between body condition measured in various ways and the individual fitness (represented for example by reproductive success) in future studies.

Another problem for assessing body condition in insects is using an appropriate control for structural body size. Common practice in most studies is to use only one structural size measurement representing total structural body size (Bommarco 1998a; Östman 2005; Öberg 2009). In beetles, body size is typically assessed based on the length of the elytron (Östman 2005; Frank et al. 2007). The results of our study suggest that one measurement is not enough to assess structural body size precisely due to morphological variability among individuals (for two specimens with the same length, one could be thin and the other one wide). However, in our study, the results of body condition analyses were only slightly affected by the various structural body size measures used. This is probably due to a quite tight correlation among structural size measurements (length of elytron, width of pronotum, length of hind femur; r-value between 0.72 and 0.78). Interestingly, the best single measure of structural body size in *P. cupreus* was the length of the hind femur, and the worst measure was the commonly used length of elytron. In contrast to other insect orders (e.g., Diptera; Blanckenhorn & Hosken 2003), length of legs is rarely used in studies on body condition in beetles, although leg dimensions and shape have been intensively studied in context of their functionality in carabids (Forsythe 1983).

The species investigated in our study is a typical polyphagous, carnivorous predator; however, there are species with alternative diets among carabids (e.g., seed predators or species specialized on a narrow spectrum of animal prey; Lövei and Sunderland 1996), so *P. cupreus* is not a universal model species that can be used for all carabid beetles. It is important to note that the significance of body condition for realized fecundity and appropriate measures of body condition may vary among species, depending on their physiology (e.g., efficiency of conversion and feeding habits). However, little is known about this topic at present, and future research is needed.

In conclusion, the use of fat content (obtained by direct fat extraction) is recommended for assessing the body condition of insects, as long as no experimental evidence exists supporting the use of dry weight or fresh weight for a given taxon. The results of this study suggest that neither dry weight nor fresh weight are able to produce results comparable to fat content (at least for *P. cupreus*). As fat extraction is a time-consuming procedure, dry weight or even fresh weight are frequently used as surrogates of fitness without any verification. Therefore, studies investigating the relationship between fitness and particular physiological parameters (e.g., fresh weight, dry weight, fat content) are sorely needed for a wide range of taxa. To prevent possible confusion in terminology, it is recommended that the use of the term “body condition” be appended with the method used, e.g., “body condition based on fat content.””
